# Supplementary material for: Association between gait speed deterioration and EEG abnormalities
Source: PLoS One. 2024 Jun 4;19(6):e0305074. doi: 10.1371/journal.pone.0305074 (PMC11149873; doi:10.1371/journal.pone.0305074)
Supplement: Attachment — Submitted filename: Reply2Reviewer1.pdf [file pone.0305074.s001.pdf]

Birmingham, 12 December 2022

Dear PLOS ONE editorial office:

Please, find below a point-to-point reply to Reviewer#1's comments.

Sincerely yours,

Valia Rodriguez-Rodriguez

Aston University

**Reply to Reviewer #1:**

Comment 1. *Some clarification is required regarding the naming of the groups used in the manuscript. Lines 158-159: "However, changes in beta band power were similar in the two groups, with 7% and 6% of participants in NorGS and B, respectively, showing energy increment in this band." NorGS was defined before and is clear, however, "group B" seems a bit unclear. If it refers to the figure1, panel B, then the samples presented in panel B should be defined somewhere in the text as group B.*

We used A and B to name the groups in the first draft. For clarity, we renamed A as NorGS (normal gait speed) and B as LowGS (low gait speed). However, we did not replace the 'B' in Lines 158-159. Thanks to the reviewer for spotting that error that is now corrected.

Comment 2: *in lines 172-173: "The difference was obtained by subtracting the spectral power of NorGS from LowGS (i.e., B-A). "Meaning that "B" refers to LowGS. Is it the same "B" as before?*

Yes, 'B' refers to 'LowGS'. As before, we did not replace it. It is corrected and lines 173-174 now read:

'The difference was obtained by subtracting the spectral power of NorGS from LowGS (i.e., LowGS - NorGS).'

<u>Comment 3:</u> *“Conclusions” section is missing. Some conclusions seem to be drawn in the Discussion section. I recommend separating the two into Discussion and Conclusions sections*

We did not include Conclusions because the section is optional according to PLOS submission guidelines. Nevertheless, we are now closing the manuscript with a Conclusion paragraph.
