# Supplementary material for: Association between gait speed deterioration and EEG abnormalities
Source: PLoS One. 2024 Jun 4;19(6):e0305074. doi: 10.1371/journal.pone.0305074 (PMC11149873; doi:10.1371/journal.pone.0305074)
Supplement: Attachment — Submitted filename: comments.docx [file pone.0305074.s002.docx]

**Hidden decline in older adults: association between gait speed deterioration and EEG**

**Abnormalities**

This work explores the gait speed and cognitive status (through MMSE) of 95 older adults. Along with this, a quantitative analysis of their resting state EEG was performed. Results showed that 70% of the sample size had lower gait speed of which 80% have abnormal EEG frequency composition. Overall, the work is fine. However, there are several important points which should be addressed before the final acceptance of the article.

1. The introduction may benefit by highlighting the relevance of using EEG modality.
2. Please cite the previous literature in the introduction which proved that resting state EEG can predict the changes in brain functions while executing the task.
3. Please add a picture of the representative participant while conducting the experiments in the method section. This will improve readability.
4. It would be better if the authors could add a flowchart describing the analysis and data processing of the study.
5. Line 101-102- “Participants were asked to close and open their eyes at different moments to explore reactivity and avoid drowsiness”. I believe that this might have affected the resting state EEG data. Additionally, the authors need to explain why they have not considered the data in eyes open condition. During the trials, the individuals were walking with their eyes open. The authors should consider this.
6. Topographical maps of Beta band are missing in figure 2. Please check.
7. Line 112-113- “This transformation expresses the distance between an individual BBSP and the average BBSP of the normal population for the participant's age.” When did the authors recruit age matched normal population? It is not mentioned in the document anywhere.
8. It will be good if the authors can discuss why they have not measured the participant’s EEG while they were walking at a certain gait speed. Measuring simultaneous EEG and gait speed would be more beneficial. It can help scientists and clinicians to interpret the hidden changes in brain activity more precisely.
9. Along with gait speed, it would be good if the authors could measure other gait parameters such as swing/ stance interval, toe-off and heel strikes, etc. and link these parameters with the changes in the cortical activity. This will add new insights into the study and help the future studies working on the rehabilitation of these individuals.
10. The authors can also consider finding the functional connectivity between highlighted brain regions.
11. The translation of the present findings into clinical applications is not clear. The article would benefit if the authors could explain this point in the conclusion of the study.
