# Supplementary material for: Association between gait speed deterioration and EEG abnormalities
Source: PLoS One. 2024 Jun 4;19(6):e0305074. doi: 10.1371/journal.pone.0305074 (PMC11149873; doi:10.1371/journal.pone.0305074)
Supplement: Attachment — Submitted filename: Reply2Reviewers.docx [file pone.0305074.s003.docx]

Birmingham, 07 January 2024

Dear PLOS ONE editorial office:

Please find below a point-to-point reply to the reviewers. We are uploading two revised versions of the manuscript: one with the track changes and another clean, together with the figures and tables.

Sincerely yours,

Valia Rodriguez – corresponding author

Aston University

**Reply to Reviewer #2:**

We are very grateful for your comments and suggestions as they have helped us improve our manuscript. Please find below a point-to-point reply to your comments.

1. The introduction may benefit by highlighting the relevance of using EEG modality.
2. Please cite the previous literature in the introduction which proved that resting state EEG can predict the changes in brain functions while executing the task.

We added the following text in the introduction:

*Given its high sensitivity, EEG is a valuable tool for assessing alterations in cerebral activity associated with functional states and for capturing the dynamic fluctuations linked to cognitive processes, emotions, and neurological conditions (12,13). EEG measurements taken during resting states hold predictive value for cognitive task performance (14,15), as the signal mirrors the functioning of underlying networks.*

3. Please add a picture of the representative participant while conducting the experiments in the method section. This will improve readability.

Gait speed (GS) assessment was not experimental, but we used a method commonly employed by physiotherapists and geriatricians in the clinical practice. Participants were instructed to stand still behind a starting line marked on the floor (that was the beginning of the acceleration zone; see diagram below) and then walk at their usual pace for 6 meters. The first and last meters were used as acceleration and deceleration zones (represented as black rectangles in the diagram), while the middle four meters constituted the testing zone (represented as a white rectangle in the diagram). GS was quantified by the researcher with a stopwatch as the time spent to cover the testing zone (i.e., 4 meters) and was expressed in meters per second (m/sec).

We added a diagram (Fig. 1), as suggested by the reviewer, with a brief explanation that reads:

*Fig 1. Diagram representing the gait speed assessment. Measurements were performed in a flat and unobstructed space by a trained researcher. Participants were instructed to stand at the beginning of the acceleration zone (first black rectangle covering 1 metre) and upon a command, to walk at their usual pace, passing the deceleration zone (second black rectangle also covering 1 metre). Gait speed was timed with a stopwatch in the middle 4 metres (time zone, the white rectangle in the diagram).*

4. It would be better if the authors could add a flowchart describing the analysis and data processing of the study.

The Fig1S -showing the analysis and data processing flow- is now available as supporting material.

5. Line 101-102- "Participants were asked to close and open their eyes at different moments to explore reactivity and avoid drowsiness". I believe that this might have affected the resting state EEG data. Additionally, the authors need to explain why they have not considered the data in eyes open condition. During the trials, the individuals were walking with their eyes open. The authors should consider this.

The reviewer correctly assumes that the resting state EEG will differ under eyes closed (EC) and eyes open (EO) conditions. It is known that the frequency composition of the EEG is different under each of these states. Furthermore, Wei et al (2018) found that while brain activity during EC was higher in sensorimotor areas and temporal cortex, there was more activity in posterior areas, including the occipital and parietal cortex during EO. Therefore, the spontaneous fluctuation of brain activity is closely related to EC and EO resting state.

However, EO was only employed in our study as a procedure to guarantee the participant kept awake during the whole recording. Consequently, we did not acquire enough time under EO to perform a frequency analysis. We preferred to acquire the EEG under EC because it is an excellent condition to study brain activity without the influence of visual stimulus driving participants' internal state – as a consequence of internal thoughts triggered by visual analysis and attention. This was especially relevant in our study as we did not have an experimental task that required a baseline at rest with EO.

Another reason behind our decision is that clinical EEG, and most of the literature that have assessed changes in the EEG frequency composition in relation to cognitive status, have used segments during EC. This state enabled us to compare our results with previous findings.

Nevertheless, we will seriously consider the reviewer's concern and include EO as a state of interest in our future studies.

- *Wei Jie et at. 2018. Eyes-Open and Eyes-Closed Resting States With Opposite Brain Activity in Sensorimotor and Occipital Regions: Multidimensional Evidences From Machine Learning Perspective. Frontiers in Human Neuroscience, 12.* [*https://doi.org/10.3389/fnhum.2018.00422*](https://doi.org/10.3389/fnhum.2018.00422)*.*

6. Topographical maps of Beta band are missing in figure 2. Please check.

Since the main changes we described in our study comprise delta, theta and alpha bands, we initially presented topographical maps for those frequency bands. We also split the alpha band into lower and higher components to compare against the literature. However, for consistency, we now produced a figure with all frequency bands, including beta and alpha bands from 8-12Hz - as used in our study. We also modified the representation, and instead of surface maps, we are showing results on volumetric maps for better visualisation.

7. Line 112-113- "This transformation expresses the distance between an individual BBSP and the average BBSP of the normal population for the participant's age." When did the authors recruit age matched normal population? It is not mentioned in the document anywhere.

We did not recruit age-matched controls. Instead, we use the normative database of the Cuban population to perform the comparison according to the participant's age. The database (Valdes-Sosa PA et al, 1990) contains quantitative EEG measures obtained from 211 healthy participants between 5 and 97 years old.

In the Methods section, we specify the following:

'… Absolute Power (AP) in the four classic frequency bands (delta, theta, alpha and beta) - here referred to as broad-band spectral parameters (BBSP) - were calculated and compared to the Cuban normative data of quantitative EEG (qEEG) [13].

The Z-transformed statistic was used to compare each participant's BBSP against the normative data.'

- *Valdes-Sosa PA, Biscay R, Galan L, Bosch J, Szava S, Virues T. High resolution spectral EEG norms for topography. Brain Topography. 1990;3:281–2.*

8. It will be good if the authors can discuss why they have not measured the participant's EEG while they were walking at a certain gait speed. Measuring simultaneous EEG and gait speed would be more beneficial. It can help scientists and clinicians to interpret the hidden changes in brain activity more precisely.

We agree with the reviewer that recording the EEG while the participant is walking is essential to understand the brain dynamics related to the implementation and execution of gait and identify specific electrophysiological changes related to deterioration in waking. Unfortunately, we did not have the necessary technology (eg. portable amplifiers that the participant could carry while walking) to perform this measurement when we conducted this study.

9. Along with gait speed, it would be good if the authors could measure other gait parameters such as swing/ stance interval, toe-off and heel strikes, etc. and link these parameters with the changes in the cortical activity. This will add new insights into the study and help the future studies working on the rehabilitation of these individuals.

We agree with the reviewer that measuring other gait parameters is also essential as they change with age, and their deterioration has been found to be associated with frailty. For instance, Montero-Odasso and co-workers (2011) found that frail older adults, besides a reduced gait speed, presented a high variability in the stride time. However, the assessment of stride time (ms), cadence (steps/min), step width (cm), and double support time (ms) require a technology (e.g. GAITRite) we did not have at the time we performed this study.

On the contrary, gait speed was a parameter easy to measure in our conditions. The latter it is also one of the points of our study: to support, beyond research, the inclusion of gait speed evaluation in the standard clinical -geriatric- assessment, given its simplicity, sensitivity and association with underlying abnormal brain function.

10. The authors can also consider finding the functional connectivity between highlighted brain regions.

We sincerely appreciate the reviewer's suggestion. We will conduct a functional connectivity analysis with our data looking at the default mode regions and the sensorimotor regions. However, we wanted to include in this paper only measurements that can be performed in the clinical practice to reach a clinical audience while reserving more 'sophisticated' analysis for another type of paper.

11. The translation of the present findings into clinical applications is not clear. The article would benefit if the authors could explain this point in the conclusion of the study.

We believe our findings can be translated into clinical practice by incorporating walking speed screening in all older adults, even the healthiest. This strategy would be beneficial to intervene in the deterioration of gait early enough and avoid falls or the slow evolution towards physical and cognitive decline. Furthermore, incorporating quantitative EEG into the specialised assessment of mobility function and rehabilitation pathways would allow risk stratification and objective evaluation of intervention success.

We have added a conclusion section were we state the following:

*Our results support screening for and intervening in early decline in mobility to prevent or delay the onset of disability and dependence. Here, we showed that, despite being physically active, older people can present declines in GS (=<0.8 m/s). Slower GS was associated in this study with abnormally slow brain electrical activity for age, representing mild brain dysfunction. The latter poses an additional risk of developing further decline, including cognitive deterioration. Electrophysiological markers such as those used in this study are inexpensive and easy to implement, which makes it feasible to incorporate them in the functional assessment of older adults. With the challenge of prolonging years of functional life in ageing societies, it is essential to design strategies to restore early declines. A strategy that combines markers such as gait speed and quantitative EEG measures may be especially valuable in objectively assessing the success of intervention programs aimed at rehabilitating changes in mobility*.

**Reply to Reviewer #3**

First, we sincerely appreciate your comments and suggestions as they have helped us improve our manuscript. Please find below a point-to-point reply to your comments.

1- Line 27: Please consider replacing "doctors" with "allied health professionals".

We agree with the reviewer that allied health professionals should be also included as some of them evaluate older adults through different pathways. However, we want to stress that doctors (i.e., physicians such as GPs and Geriatricians) also overlooked early changes as they do not proactively assess older people function to detect subtle modifications. We feel that the term' health professional' includes all categories. Therefore, we changed the sentence, and now it reads:

*These changes, frequently overlooked by patients and health professionals…*

2- Lines 32-33: The information regarding gait speed was not clear in the abstract. "We measured their gait speed at the usual pace (0.8 m/s cut-off point)". In lines 35-36, the authors stated that 70% of the sample had a preclinical gait speed deterioration (i.e. lower than 0.8 m/s). After all, was 0.8 m/s seen as a sharp cut-off value for the usual pace or as an impaired gait speed? The abstract does not make this clear. Please make this information more understandable. I understand you, firstly, just measured walking speed. After, you consider the value of 0.8 m/s to differentiate the groups based on participants' gait speed.

We agree with the reviewer that the sentence is not clear. We modified it, and now it reads:

*We measured their gait speed at the usual pace and the cognitive status using the MMSE. A value of 0.8 m/s was used as a cut-off point to decide whether they presented a decline in gait speed.*

3- Lines 62-63: Please provide at least a reference to this statement: "However, gait is a process that, besides musculoskeletal and sensorial components, also depends on significant contributions from high-order cerebral areas for planning, execution, and control."

The following citations were added:

- *Allali G et al (2019). Brain Structure Covariance Associated With Gait Control in Aging. J Gerontol A Biol Sci Med Sci . 74(5):705–13.*
- *Yogev-Seligmann G, Hausdorff JM, Giladi N. (2008). Movement Disorders. 28:329–42.*
- *Holtzer R, Verghese J, Xue X, Lipton RB (2006). Neuropsychology. 20(2):215–23.*

4- Lines 63-66: Please provide at least a reference to this statement: "A preclinical deterioration of GS in older individuals without significant peripheral disorders or uncontrolled systemic disease is likely to be accompanied by subclinical changes in brain function as part of its underlying physiopathology."

The following citations were added:

- *Wilson J et al (2019). Neuroscience and Biobehavioral Reviews. Elsevier Ltd; p. 344–69.*
- *Verlinden VJA et al (2014). Alzheimer's and Dementia. 10(3):328–35.*
- *Holtzer R et al (2014). J Gerontol A Biol Sci Med Sci. 69(11):1375–88*.

However, we also modified the sentence, and now it reads:

*"deterioration of GS in older individuals that cannot be attributed to significant peripheral disorders or uncontrolled systemic disease is likely to be associated with abnormal brain function as part of its underlying physiopathology."*

5- Methods

Lines 90, and 117: Please standardise the gait unit measure to m/s.

Done

6- Why did the authors opt for dichotomous data (GS slower than 0.8 m/s: yes or no; normal or abnormal BBSP) and a Chi-square test, rather than continuous data for correlation analyses, for example, by a Pearson or Spearman correlation test?

We chose a Chi-square test because while the GS data were continuous, the decision of whether the EEG was normal or not was dichotomous. That is, the participants' quantitative EEG data was classified as abnormal if the absolute Z value of the absolute power for any frequency band was above 1.96; otherwise it was consider as normal. Therefore, we also categorised the GS data using the study cut-off point to assess the association's presence.

Nevertheless, considering the reviewer's suggestion, we have now performed a logistic regression using the GS value as a continuous predictor of abnormality in the EEG. The results showed that GS predicts whether the EEG is abnormal or not (Wald test (1)=8.6, p=0.003; OR= 2.96, CI: 1.05,5.3). This additional test was added to the Method and Results section of the manuscript.

7- Results

Table 1 – Please replace "N" with "n".

Done

8- Table 1 – Please delete the comma in "Mean (SD),".

Done

9- Discussion

Were the study hypotheses confirmed or refuted?

Our hypothesis was that a GS decline in our participants would be associated with a low MMSE score (<25 ) and EEG abnormal for the age. We found that 54 of 63 individuals with GS decline also had a significantly slower EEG. However, ONLY 10 of the 54 older adults with reduced GS and slow EEG had a MMSE < 25. Therefore, our hypothesis was partially confirmed.

10- Despite the participants age around 77 years old, living independently in the community, and practicing physical activity with moderate regularity, to what do the authors attribute the percentage of 70% of the sample to have presented walking speed below 0.8 m/s? This is a relatively low gait speed for community-dwelling older adults with a relatively good level of physical activity. Please address this issue in the discussion, based on the literature.

We agree with the reviewer and discussed this finding in the discussion section. Specifically, we added the following text:

*Even though participants in our study engaged in regular, mild exercise in the community, the GS decline was present in 70% of the sample. The fact that both groups were mainly composed of women with a mean age of 76.8 (NorGS) and 80 (LowGS) could have been one of the reasons for the predominance of low GS values. While there was not a significant difference in age between groups, it is known that the level of physical deterioration increases with age. Muscle strength decreases by 1.5% per year and accelerates to 3% per year after 60 years of age(Amrya et al, 2018). Therefore, the combination of a higher female composition and a mean age of about 80 could have determined lower GS values in the LowGS group. Nevetheless, the prevalence of lower values compared to the literature is puzzling. Previous studies conducted in comparable samples have found higher GS values. For example, in their 2022 study, Dommershuijsen and colleagues found that individuals aged 89 exhibited GS values higher than 1 m/s for both sexes, while men and women older than 90 showed GS values of 0.9 m/s and 0.8 m/s, respectively. Bohannon and Wang (2019) reported similar findings, with the exception that GS for women aged 80-85 was 0.88 m/s. A study by Lau and co-workers. (2020) described normative values higher than 1 m/s in Southeast Asian older adults of both sexes aged below 80 and 0.8 m/s in participants aged 81 and older. Finally, Lusardi's study also found that older women aged 80 and above showed GS value of 0.8 m/s (SD 0.20).*

*One explanation for the disagreement with the literature could be methodological differences in the way GS was measured. While Bohannon and Wang (2019) assessed the gait speed in 4 m walkway, they did not use an acceleration zone. Different studies have reported that dynamic or static protocols produce different results (Krumpoch et al., 2021; Macfarlane PA et al., 2008; Phan et al., 2012). On the other hand, Dommershuijsen, Lau and Lusardi's studies measured the participants's gait with a GaitRite system and 6 m/s walkways. Short (3 – 4 m) and long ( 5 – 10 m) walkway lengths produce reliable results across testing sessions but they do not have sufficient concurrent validity (Peters et al., 2013). The same applies to gait measurements obtained with the GaitRite system and walking tests using a stopwatch. In a study by Peters and co-workers (2014), GS measured with GaitRite showed higher values in all trials performed by community ambulators than the 3 m walking test.*

*We cannot ignore, however, the socioeconomic characteristics of the study sample. Despite being classified as an upper middle-income country according to the World Bank, Cuba's sustained poor economic situation puts individuals in constant hardships. Notwithstanding good health care and social initiatives to improve life, older people are one of the main vulnerable groups affected by pensions of deficient purchasing power, food scarcity and lack of variety, deficient nutrition, deteriorated housing, uncertainty regarding the future and persistent stress. We cannot rule out that these conditions have also influenced our results as it is known that wider determinants, nutrition and well-being impact people's health, including their frailty levels and how they age.*

- Amarya S, Singh K and Sabharwal M (2018). Ageing Process and Physiological Changes. Gerontology. InTech. DOI: 10.5772/intechopen.76249
- Bohannon RW and Wang YC (2019). Four-Meter Gait Speed: Normative Values and Reliability Determined for Adults Participating in the NIH Toolbox Study. Arch Phys Med Rehab 100(3):509-13
- Dommershuijsen LJ, Ragunathan J, Ruiter R, Groothof D, Mattace-Raso FUS, Ikram MA, et al. Gait speed reference values in community-dwelling older adults – Cross-sectional analysis from the Rotterdam Study. Exp Gerontol [Internet]. 2022 Feb 1 [cited 2022 Feb 24];158:111646. Available from: https://linkinghub.elsevier.com/retrieve/pii/S0531556521004289
- Krumpoch S, Lindemann · Ulrich, Rappl A, Clemens Becker ·, Cornel ·, Sieber C, et al. The effect of different test protocols and walking distances on gait speed in older persons. 2021;33:141–6.
- Lau LK, Wee SL, Pang WJB, Chen KK, Abdul Jabbar K, Yap PLK, Mallya JU, Ng DHM, Tan QLL, Seah WT, Ng TP. Reference Values of Gait Speed and Gait Spatiotemporal Parameters for a South East Asian Population: The Yishun Study. Clin Interv Aging. 2020;15:1753-1765.
- Lusardi MM, Pellecchia GL, Schulman M. Functional Performance in Community Living Older Adults. Journal of Geriatric Physical Therapy [Internet]. 2003. 26(3):14–22.
- Macfarlane PA, Looney MA. Walkway length determination for steady state walking in young and older adults. Res Q Exerc Sport. 2008;79(2):261–7.
- Phan-Ba R, Calay P, Grodent P, Delrue G, Lommers E, Delvaux V, et al. A corrected version of the Timed-25 Foot Walk Test with a dynamic start to capture the maximum ambulation speed in multiple sclerosis patients. NeuroRehabilitation. 2012;30(4):261–6.
- Peters DM, Fritz SL, Krotish DE. Assessing the reliability and validity of a shorter walk test compared with the 10-Meter Walk Test for measurements of gait speed in healthy, older adults. J Geriatr Phys Ther. 2013 36(1):24–30.
- Peters DM, Middleton A, Donley JW, Blanck EL, Fritz SL. Concurrent validity of walking speed values calculated via the GAITRite electronic walkway and 3 meter walk test in the chronic stroke population. Physiother Theory Pract. 2014;30(3):183–8.

11- Line 204: The authors stated, "The association between an early decline in gait speed and an abnormal EEG is not surprising." So what was the additional motivation for this study if this result was already expected? Also, what do the authors consider an "early decline in gait speed"? Older adults with not very advanced ages or individuals with a walking speed that is not very compromised, i.e., above 1 m/s?

In our study, we assumed that, given the complexity of gait implementation and control, if an older adult presents with a gait speed lower than the expected for the age – not explained by musculoskeletal disorders- she/he must have had an associated brain dysfunction measurable with EEG. However, our assumption needed to be tested, which was our study's motivation.

We consider that an early decline in gait speed is a change in walking speed presented by ambulators and functionally independent individuals. An older individual with an early decline in gait speed will not have a mobility disability, and walking could seem apparently 'normal' for the age.

12- Lines 225-227: What do the authors consider "preclinical GS deterioration"? I don't consider a GS below 0.8 m/s as a preclinical characteristic. This is a very considerable and evident gait impairment. Also, "hidden changes in GS". What do you attribute this term to? GS has been objectively evaluated and has nothing hidden chance. The GS, for my understanding, is very evident. Perhaps hidden changes in electroencephalographic activity and not in GS itself.

By preclinical, we meant that an individual with 0.8 m/s while at risk of falls, hospitalisations and cognitive decline years later, will not present yet with disabilities. I think detecting that an older adult has an evident gait impairment without measuring it when the speed is around 0.8 m/s will depend on the observer's expertise in assessing older people.

However, the point we want to make in our paper is that screening for early gait declines in apparently healthy older individuals is essential. By 'early', we mean a decline that has not evolved yet to become a disability, and therefore, it should respond better to rehabilitation interventions. To avoid misunderstanding, we eliminated the term 'preclinical gait decline' and used instead gait decline or early decline.

13- Lines 227-229: The authors stated "Different studies have reported, however, that MMSE is not a sensitive tool for detecting minor cognitive impairment [28,29]." Given that, why did the authors choose to use the MMSE instead of MoCA, for example? So, from what I understand, the authors wanted to verify the ability of the MMSE to identify any "preclinical GS deterioration". If the authors already knew from the literature that the MMSE is not a sensitive tool for detecting minor cognitive impairment, why, even so, did they use this instrument in the study?

When this study was conducted, MMSE was the tool used in our research. In recent studies, we have incorporated MOCA and neuropsychological tests that correlate with gait speed.

14- Lines 230-231: The authors stated "Another possibility to consider is that our participants were in an early stage where cognitive changes had not yet occurred." Was it the MMSE test that failed to identify the participants' minor cognitive impairment, or were they actually in this stage of the condition? Perhaps the authors would have had a more accurate tool for identifying mild cognitive impairment if they had employed MoCA, for instance. Due to the potential for a false negative generated by the instrument employed to detect mild cognitive impairment, the authors should take another look at this lack of relationship.

We agree with the reviewer. The use of MMSE was a limitation in this study – we acknowledge this in the discussion. In the second phase of this study, where we are re-assessing all individuals that are still alive and also new volunteers, we have incorporated MOCA int the evaluation of cognitive status.

15- Line 242: "Preclinical GS decline was present in 70% of the sample". I continue to believe that a gait speed of less than or equal to 0.8 m/s should not be regarded as a preclinical characteristic. According to the research, this is already a significantly reduced walking speed.

We have eliminated the phrase 'preclinical GS decline' from the manuscript. We believe that the results of our study also support that a GS less than or equal to 0.8 m/s should not be dismissed.
