# Supplementary material for: Association between gait speed deterioration and EEG abnormalities
Source: PLoS One. 2024 Jun 4;19(6):e0305074. doi: 10.1371/journal.pone.0305074 (PMC11149873; doi:10.1371/journal.pone.0305074)
Supplement: Attachment — Submitted filename: Comments R2.docx [file pone.0305074.s004.docx]

**Association between gait speed deterioration and EEG Abnormalities**

**Minor revision**

1. Flowchart describing the analysis and data processing of the study should be included in the article, not as a supporting information.
2. Measuring simultaneous EEG and gait parameters can help scientists and clinicians to interpret the hidden changes in brain activity more precisely. Also, the association between the two can be done more accurately when the data is recorded in synchronization.

I am not really satisfied with the author response on this. I believe the study's protocols has limitations.

If the authors can discuss this limitation in detail, it will be good for future research in this field.
