# Supplementary material for: Association between gait speed deterioration and EEG abnormalities
Source: PLoS One. 2024 Jun 4;19(6):e0305074. doi: 10.1371/journal.pone.0305074 (PMC11149873; doi:10.1371/journal.pone.0305074)
Supplement: Attachment — Submitted filename: Reply to reviewers.docx [file pone.0305074.s005.docx]

**Association between gait speed deterioration and EEG Abnormalities**

**Point-to-point reply to the reviewers.**

Reviewer 1

Reviewer #1: Although authors states that the dataset is available from the Aston Data Explorer, I was unable to locate it. Sharing a link or some guidance on how to access it would be helpful. I don't have any additional comments regarding the manuscript.

Reply: Some information was lacking in the dataset metadata and as a consequence, it was not properly shared. This is now fixed, and the dataset is available here:

<https://doi.org/10.17036/researchdata.aston.ac.uk.00000557>.

Reviewer 2

1. Flowchart describing the analysis and data processing of the study should be included in the article, not as a supporting information.

Reply: The flowchart is now included as Figure 2. The rest of the figures were re-numbered.

2. Measuring simultaneous EEG and gait parameters can help scientists and clinicians to interpret the hidden changes in brain activity more precisely. Also, the association between the two can be done more accurately when the data is recorded in synchronization.

I am not really satisfied with the author response on this. I believe the study's protocols has limitations. If the authors can discuss this limitation in detail, it will be good for future research in this field.

Reply: The following paragraph was added acknowledging the benefits of simultaneous acquisition of EEG and gait:

*In this study we assessed the association between gait speed decline and the presence of brain dysfunction by analysing the EEG at rest. However, we aim to incorporate in future research studies, the simultaneous acquisition of brain electrical activity during walking tasks which was not possible in this investigation. By simultaneously acquiring the EEG and gait, it is possible to elucidate specific changes in brain dynamics related to the different phases of gait. Furthermore, the high temporal resolution of EEG allows us to closely follow the coupling between movement planning, implementation and control and the brain's electrical activity. This strategy is essential to better understand the nature of the gait deterioration and design specific rehabilitation.*
